# Research on Tea Tree Growth Monitoring Model Using Soil Information

**DOI:** 10.3390/plants11030262

**Published:** 2022-01-19

**Authors:** Ying Huang, Hao Jiang, Weixing Wang

**Affiliations:** 1 Electronic Information School, Wuhan University, Wuhan 430072, China; huangying800816@163.com; 2 School of Automatic Control, Liuzhou Railway Vocational Technical College, Liuzhou 545616, China; 3 School of Electronic Engineering, South China Agricultural University, Guangzhou 510642, China

**Keywords:** crop growth model, soil indicators, normalized difference vegetation index (NDVI), long short-term memory (LSTM)

## Abstract

Crop growth monitoring is an important component of agricultural information, and suitable soil temperature (ST), soil moisture content (SMC) and soil electrical conductivity (SEC) play a key role in crop growth. Real-time monitoring of the three soil parameters to predict the growth of tea plantation helps tea trees grow healthily and to accurately grasp the growth trend of tea trees. In this paper, five different models based on the polynomial model and power model were used to construct the soil temperature, soil water content and soil conductivity and tea plantation growth monitoring models. Experiments proved that tea plantation growth were positively correlated with ST and negatively correlated with SMC and SEC, and among the constructed models, the ternary cubic polynomial model was the best, and R square (R^2^) of the constructed models were 0.6369, 0.4510 and 0.5784, respectively, indicating that SEC was the most relevant to tea plantation growth maximum. To improve the prediction accuracy, a model based on sum of soil temperature (SST), sum of soil water content (SSMC) and sum of soil conductivity (SSEC) was proposed, and the experiments also showed that the ternary cubic polynomial model was the best, with 0.9638, 0.9733 and 0.9660, respectively. At the same time, a model incorporating three parameters such as soil temperature, soil water content and soil conductivity was also suggested, with 0.6605 and 0.9761, respectively, which effectively improved the prediction accuracy. Validation experiments were conducted. Twelve data sets were utilized to verify the performance of the model. The experiments showed that the regressions in the polynomial models achieved a better prediction effect. Finally, a long short-term memory (LSTM) network prediction model optimized by the bald eagle search algorithm (BES) was also constructed, and R^2^, root mean square error (RMSE), mean squared error (MSE), mean absolute error (MAE) and mean absolute percentage error (MAPE) of prediction were 0.8666, 0.0629, 0.0040, 0.0436 and 10.5257, respectively, which significantly outperformed the LSTM network and achieved better performance. The model proposed in this paper can be used to predict the actual situation during the growing period of tea leaves, which can improve the production management of tea plantations and also provide a scientific basis for accurate tea planting and a decision basis for agricultural policy formulation, as well as provide technical support for the realization of agricultural modernization.

## 1. Introduction

Crop growth monitoring is an important element of agricultural information that provides nondestructive access to crop growth. Crop growth is often affected by various environmental factors, such as atmospheric temperature, soil temperature, soil moisture content, soil electrical conductivity, rainfall, etc. Measures to monitor the environmental parameters affecting crop growth in a timely and accurate manner can help crops grow healthily and can accurately grasp crop growth trends, which plays an important role in improving crop yields, a key element of fine agricultural management.

The ground monitoring method, crop growth modeling method and remote sensing monitoring method are the principal methods of crop growth monitoring [1]. The ground monitoring method is a direct and rapid method based on manual observation, which assesses the leaf area index (LAI) and height and density of crops by technicians and has the advantages of high accuracy and good continuity but is influenced by human subjective factors and is only suitable for small area monitoring. The growth modeling method has become one of the most powerful tools in crop growth decision making by using crop physiology, integrating the results of some advanced technologies such as examinations of atmospheric and soil factors, and highlighting their advantages. The remote sensing monitoring method, with its real-time dynamic characteristics, is helpful in monitoring crop growth on a large scale through the study of foliar indices and biomass.

Crop growth monitoring methods are mainly direct monitoring methods, image classification methods, contemporaneous comparison methods, crop growth process monitoring methods, crop growth modeling methods and diagnostic modeling methods [2,3]. LAI, net primary productivity (NPP) and normalized difference vegetation index (NDVI) are considered to be more effective indices for monitoring crop growth, among which NDVI is the most widely used, and it can dynamically reflect the process of vegetation change. Refs. [4,5,6,7,8,9] explored the relationship between NDVI and climate influences such as climate temperature and rainfall. Some studies found that the normalized NDVI has some correlation with soil moisture [10,11,12]. A positive correlation between NDVI and indices such as precipitation and potential evapotranspiration is revealed [13]. A simulation model is presented for wheat growth and yield under water and temperature stress conditions, which can well predict growth and yield [14].

In addition to the wide coverage, timeliness, high comprehensiveness and good economic characteristics of the method of crop monitoring using remote sensing, there are also some problems [15]: (1) insufficient accuracy and limitations of remote sensing satellite data for continuous real-time monitoring; (2) single structure of remote sensing data and the lack of quantitative mathematical tool models; (3) crop monitoring model analysis is too conventional and can only be applied to specific models, which has some limitations; (4) lack of intelligent process monitoring instruments or equipment; (5) insufficient cross-fertilization of remote sensing with other disciplines; (6) low monitoring effect and lack of consideration of soil parameters, meteorological parameters and other affecting factors; and (7) lack of professional and technical staff in remote sensing, which makes it difficult to form characteristic monitoring techniques, and lack of corresponding support. NDVI can be extracted from remote sensing images, which require strong expertise, and there is a delay in NDVI acquisition, so it is necessary to use neural networks or artificial intelligence algorithms to improve the accuracy of prediction [16].

The growth of crops is influenced by soil conditions and presents in different crop growth characteristics. Many scholars have looked for the relationship between soil properties and crop growth. Studies have shown that soil conductivity shows a distinct relationship with crop growth or yield, and there is no uniform relationship [17]. Suitable soil conductivity can improve soil nutrients and promote healthy crop growth [18,19,20].

Computational and learning models constructed by computer vision, machine learning and deep learning also play an important advantage in crop growth [21,22,23]. Regression modeling is used to identify maize longevity by combining LAI and MDA [24]. Computer vision technology is proposed for the maize growth monitoring method, which will be affected by the image powder rate and other environments and is difficult to apply in practice [25]. Deep local association neural network is proposed for the maize growth model and can effectively solve the problem of difficult recognition [26].

The models and methods described above were in a position to reveal the relationship between climatic parameters such as temperature, rainfall, potential evaporation volume, soil conductivity and crop growth and predicted crop growth trends through relevant algorithms. However, no similar solutions have been seen for integrating methods for predicting crop growth models with multiple influencing factors in soil.

The objectives of this paper are to study the correlation between soil moisture content, soil temperature and soil electrical conductivity and NDVI of tea plantation; to construct a prediction model based on soil moisture content, soil temperature and soil electrical conductivity with NDVI; to apply the LSTM network model optimized by BES to predict the growth of tea plantation; and to verify the performance of the prediction model.

Section 1 introduces the crop growth monitoring methods and the current status of their research; Section 2 describes the research methodology as well as the data acquisition methods; Section 3 constructs the prediction models based on oil moisture content, soil temperature and soil electrical conductivity with NDVI and their experimental analysis; Section 4 constructs the models and analysis based on LSTM networks optimized by BES; finally, conclusions and discussion are presented.

## 2. Research Methodology

### 2.1. Study Area and Subject

Liucheng County, Liuzhou city, Guangxi province, which is in the subtropical monsoon zone, has the advantages of suitable temperature, sufficient light and abundant annual average rainfall, but there exists an uneven distribution of quarters or months, which often causes seasonal water shortage. The average temperature of Liucheng County is around 20 °C throughout the year, and the annual rainfall is 1300–1500 mm, which is a good climatic condition for most plants. The subject of this paper is the tea plantation of Guangxi State-owned Fuhu Overseas Chinese Farm (109°21′ N, 24°82′ E), with a planting area of roughly 58.4 ha.

### 2.2. Data Acquisition

#### 2.2.1. Soil Information Data

Soil information data were collected by soil temperature sensors, soil moisture content sensors and soil electrical conductivity sensors deployed in the tea plantation. Since the tea plantations are mostly located in mountainous and sloping areas, which are often affected by obstacles and increase the communication distance and quality of wireless sensor nodes, wireless transmission and direct current (DC) line communication technology were used to realize the dual transmission of data.

MEC20 sensors produced by Dalian Zheqin Technology Co. were adopted in the paper to collect soil moisture content, soil electrical conductivity and soil temperature. Their parameters are given in Table 1.

According to the measurement requirements, the voltage output type sensor with 6 m line length is selected, and the range of SMC is 0–100%, and the range of SEC is 0–5000 μs/cm.

According to the actual area of Liuzhou Tea Garden, five sensor nodes were deployed in a distributed deployment mode, which is responsible for obtaining soil indicators for assigned areas. The sensor should be buried approximately 4–6 inches (10–15 cm) below the ground surface. For a better indication of the average humidity across the root zone, sensors can be placed at multiple depths.

Data collected from sensor nodes are sent to the gateway node through wireless transmission or DC line communication, then forwarded by the gateway node to the remote server through 4G wireless communication method, and finally stored in the database for subsequent analysis of the prediction model. The ST, SMC and SEC of the tea plantation studied in this paper were collected from 1 January 2020 to 31 December 2020, which are presented in Table 2.

#### 2.2.2. Tea Growth Data

LAI, NPP and NDVI are considered to be more effective indices for monitoring crop growth, among which NDVI is the most widely used, and it can dynamically reflect the process of vegetation change.

Sentinel-2 high-resolution multispectral imaging satellites, which cover 13 spectral bands with a maximum resolution of 10 m, are utilized in this paper. It is the one who contains three bands in the red-edge range and can effectively monitor the vegetation health. NDVI is calculated from Equation (1).

(1) $$\mathrm{NDVI}=\frac{\mathrm{IR}\;-\;R}{\mathrm{IR}+R}$$

where IR is the pixel value in the infrared band, and R is the pixel value in the red band.

**Figure 1:**
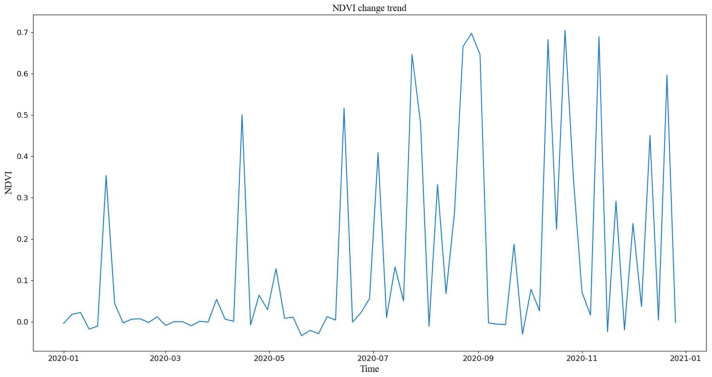
NDVI values.

NDVI values of tea plantation from 1 January 2020 to 31 December 2020, were extracted using Google Earth Engine (GEE) platform, which are shown in Figure 1.

### 2.3. Experimental Platform

Google Earth Engine (GEE) platform is utilized to extract the NDVI of tea plantations. It is a comprehensive platform for geographic information data processing and visualization launched by Google.

EDA (Exploratory Data Analysis) and HeatMap tools were utilized to analyze the correlation between multiple objects and multiple attributes. EDA allows the analyst to see at a glance the patterns implied in the data and come up with model for the data. HeatMap, as one of the most common visualization tools, is widely used in various big data analysis scenarios because of its rich color variations and vivid and detailed information expression.

Model construction and performance validation are conducted under the MATLAB platform.

## 3. Model Construction Experiments and Analysis

### 3.1. Data Processing

Remote sensing observations are relatively influenced by cloud cover and sensors, resulting in precipitous drops in NDVI values that cannot be well utilized. It can be observed from Figure 1 that NDVI does experience many distortions. NDVI values (72 groups a year) extracted by GEE are filtered to achieve the reconstruction of NDVI. The main methods of filtering are HANTS, spline interpolation, Savitzky–Golay (S–G), sliding the average method and median filtering. Considering that the characteristics of vegetation do not change abruptly, the maximum value of NDVI was selected for filtering by the S–G filtering method in order to ensure information retention of the collected data [27]. The filtered NDVI value can be obtained by Equation (2).

(2) $$Y_{i}^{*}=\sum_{i=-n}^{n}W_{j}Y_{i+j}/M$$

where *Y* is the original NDVI value, Y* is the filtered NDVI value, *i* denotes the original NDVI order, *W_j_* is the weight of the *j*th NDVI value at the beginning of the filter window, M is the window size (taking the value 2*n* + 1) and *n* is half the length of the window.

**Figure 2:**
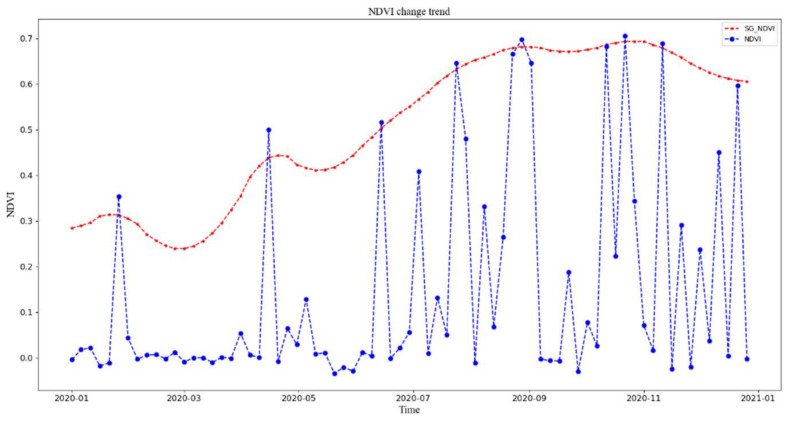
NDVI values before and after filtering.

Combined with the harvested tea leaves, the window size of the SG filter was taken as 5, the order of the fitted polynomial was taken as 2, and the standard deviation was taken as 0.01. Values before and after filtering are shown in Figure 2.

**Figure 3:**
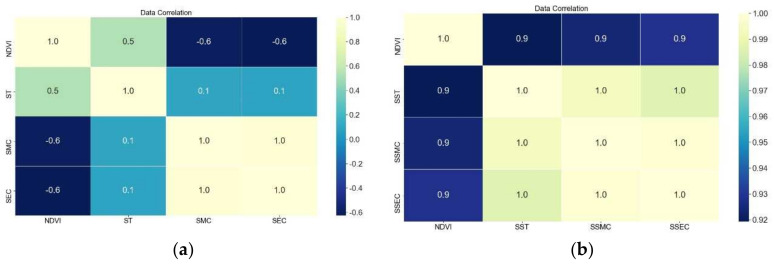
Correlation of soil information data and NDVI. (**a**) soil information data, (**b**) soil information data (Summation).

A total of 47,557 soil information data were gathered during the time period under study, and the data were normalized. The soil information data were averaged according to the time points corresponding to the collected NDVI. Sentinel-2 consists of Sentinel-2A and Sentinel-2B. Each satellite is in an imaging period of 10 days, and the two satellites complement each other for imaging, so the two satellites alternate, with a revisit period of 5 days. Thus, the average window of the data is equal the first five days of the satellite image time point. Then, correlation analysis was performed for NDVI, soil water content, soil conductivity and soil temperature, and the results are presented in Figure 3.

Figure 3a shows moderate to high correlation between the NDVI on one hand and ST, SMC and SEC on the other, both around 0.5. NDVI was significantly correlated with ST and negatively correlated with SMC and SEC.

Since ST, SMC and SEC are cumulative for crops, daily data of ST, SMC and SEC above the value of 0 were cumulative (respectively as SST, SSMC and SSEC), and correlation analysis was performed with NDVI [28]. Figure 3b shows the high correlation between the NDVI on one hand and SST, SSMC and SSEC on the other, both around 0.9, which indicates that they are under a high correlation, both being positively proportional.

### 3.2. Evaluation Indicators

Four evaluation indicators are used to evaluate the performance of the model. They are the sum of squared error (SSE), R-square (R^2^), adjusted r-square (AR) and root mean square error (RMSE). They can be calculated by Equations (3)–(6).

(3) $$\mathrm{SSE}={\sum_{i=1}^{n}\omega_{i}\left(y_{i}-\overset{\wedge }{y_{i}}\right)}^{2}$$

(4) $$\mathrm{RMSE}=\sqrt{\frac{1}{n}}\sum_{i=1}^{n}{\left(y_{i}-\overset{\wedge }{y_{i}}\right)}^{2}$$

(5) $$R^{2}=\frac{\sum_{i=1}^{n}\omega_{i}{\left(\overset{\wedge }{y_{i}}-{\overset{-}{y}}_{i}\right)}^{2}}{\sum_{i=1}^{n}\omega_{i}{\left(y_{i}-\overset{-}{y_{i}}\right)}^{2}}$$

(6) $$R^{2}=\frac{\sum_{i=1}^{n}\omega_{i}{\left(\overset{\wedge }{y_{i}}-{\overset{-}{y}}_{i}\right)}^{2}}{\sum_{i=1}^{n}\omega_{i}{\left(y_{i}-\overset{-}{y_{i}}\right)}^{2}}$$

where *n* represents the number of samples, *p* represents the number of features, $\hat{y}$ represents the predicted value, $\bar{y}$ represents mean of the measured value and *y* represents the measured value.

### 3.3. Model Construction of Tea Growth

An experimental approach is used to construct the model. First, 60 sets of 72 data sets were used for model construction, and the remaining 12 sets were used to validate the performance of the model.

#### 3.3.1. Model Prediction of Temperature and Tea Growth

**Figure 4:**
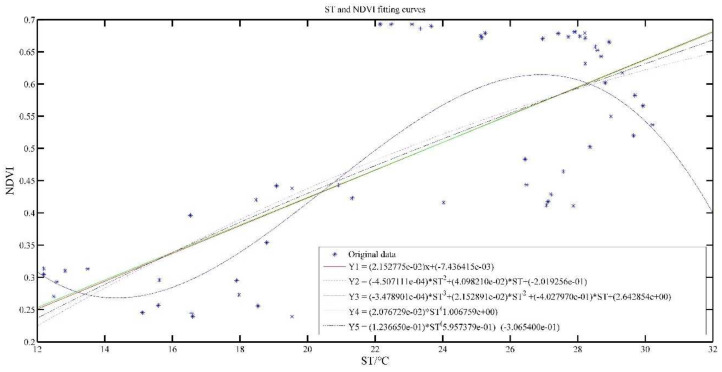
ST and NDVI model results.

**Figure 5:**
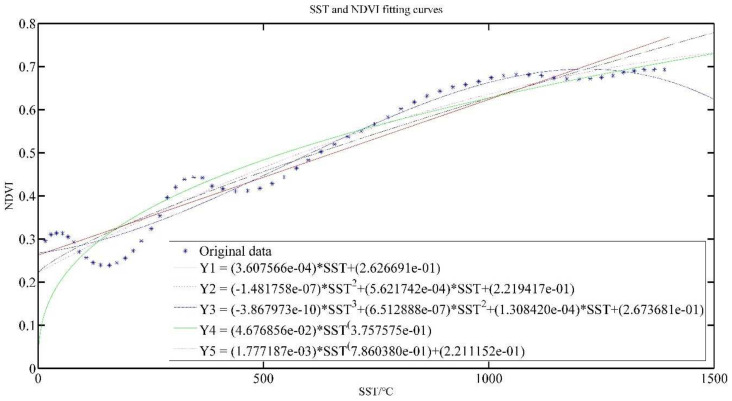
SST and NDVI model results.

Figure 4 and Figure 5 show the effect of ST and SST fitted with NDVI, respectively. The polynomial model and power model are applied for model construction, and the exponents of polynomials are chosen as 1, 2 and 3, while the number of terms in the power model is chosen as 1 and 2, and the models are named as Y1, Y2, Y3, Y4 and Y5, respectively.

Y1 models show that as the temperature increases, NDVI value increases gradually. The ST is commensurate with NDVI, which is consistent with the analysis results in Figure 3.

Table 3 presents the results of the evaluation indicators for the temperature construction model. From Table 3, we can see that the coefficients of determination are all relatively low, indicating that the correlation is low. R^2^ of Y1–Y5 ST models were only 0.5673, 0.5723, 0.6369, 0.5672 and 0.5690, respectively. The SSE, RMSE and AR of Y1–Y5 models do not differ much, but it can be observed that the Y3 model is the best.

The $R^{2}$ of Y1–Y5 SST models were 0.9286, 0.9476, 0.9638, 0.8945 and 0.9357, respectively, all around 0.8, which indicates that the model has good performance. SSE and RMSE of all models are also relatively small, indicating that the data fit dispersion is small, and the AR indicators are both above 0.8, indicating that the fitted model is better. It also can be seen that the Y3 model is the best.

It also can be seen from Figure 5 that the higher SST is in a certain range, the higher the NDVI value will be, but when the SST increases to a certain value, the NDVI value gradually decreases when the SST is larger, which is consistent with the growth of tea leaves. In the process of tea growth, the increase of temperature plays a role in promoting the growth of tea, so NDVI value will also increase; when the temperature increases further, it will lead to soil water shortage due to high temperature, and even tea roots will be burned, affecting the growth rate, or may even die [29]. This is consistent with the crop physiological process.

#### 3.3.2. Model Prediction of Soil Moisture Content and Tea Growth

**Figure 6:**
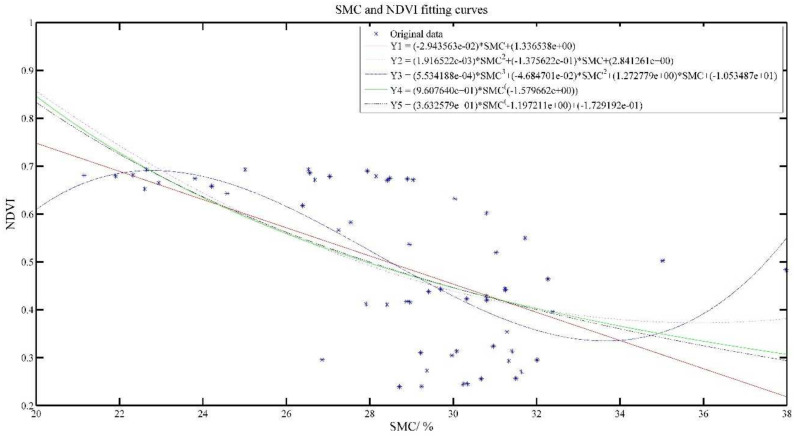
SMC and NDVI model results.

**Figure 7:**
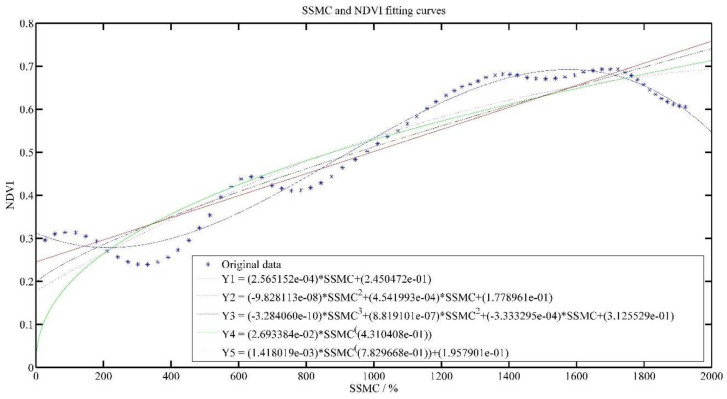
SSMC and NDVI model results.

Figure 6 and Figure 7 show the effect of SMC and SSMC fitted with NDVI, respectively.

As the SMC increased, NDVI value also gradually decreased. SMC was inversely associated with NDVI value, but the SSMC was positively related to NDVI value, which is consistent with the analysis results in Figure 3.

In Table 4, for the SMC model, R^2^ of Y1–Y5 models were only 0.3464, 0.3830, 0.4510, 0.3647 and 0.3653, respectively. The SSE, RMSE and AR of Y1–Y5 models do not differ much, but it can be seen that the Y3 model is the best. For the SSMC model, R^2^ of all models reached above 0.8, indicating that there is a positive relationship between cumulative soil water content and NDVI, and the Y3 model is the best.

In a certain range, the higher the SSMC, the higher the NDVI value, but when the SSMC increases to a certain value, the NDVI value gradually decreases as the SSMC becomes larger, which is consistent with the growth of tea. During the growth of tea, moderate moisture is especially important for tea growth. Tea will be influenced by too much or too little water. This is also in line with the crop physiological process.

#### 3.3.3. Model Prediction of Soil Electrical Conductivity and Tea Growth

**Figure 8:**
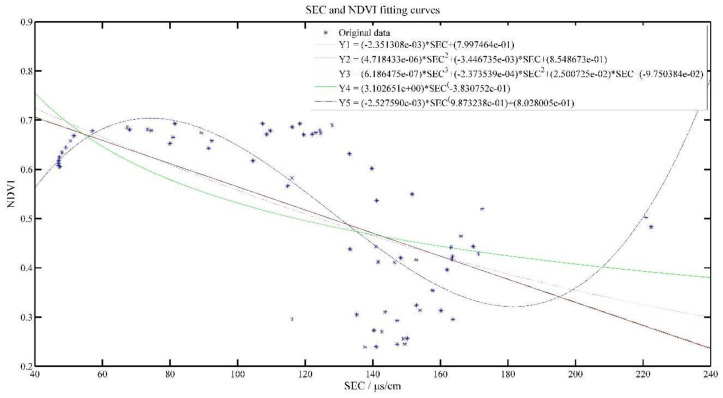
SEC and NDVI model results.

**Figure 9:**
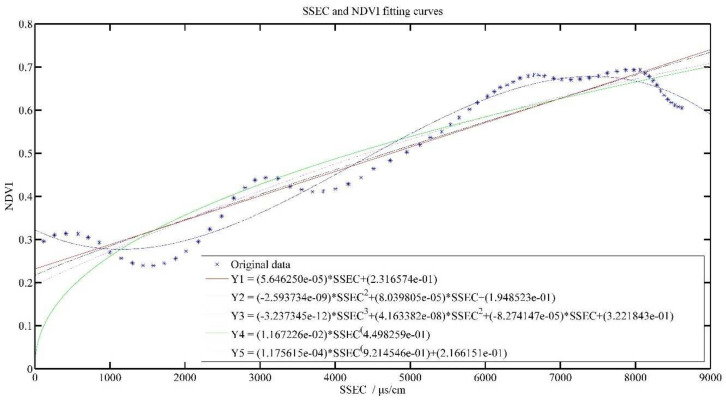
SSEC and NDVI model results.

Figure 8 and Figure 9 show the effect of SEC and SSEC fitted with NDVI value, respectively. With the increase of SEC, NDVI value gradually decreases, i.e., SEC was inversely related to NDVI value, but SSEC was positively related to NDVI value, which is consistent with the analysis results in Figure 3.

As can be seen from Table 5, Y3 model has the best R^2^ at 0.5784, while for the SSEC model, the R^2^ of all models is above 0.8, indicating a positive relationship between cumulative soil conductivity and NDVI.

Moderate SEC is an important condition. If the SEC is too high (too much salt) it will make the tea inhibit the synthesis of chlorophyll, which is not conducive to growth; if it is too low, it will make the tea not absorb enough nutrients and water, which greatly affects the growth of tea [29]. This is also in line with the physiological process of the crop.

#### 3.3.4. Model Prediction of Tea Growth with Multiparameter Fusion

**Figure 10:**
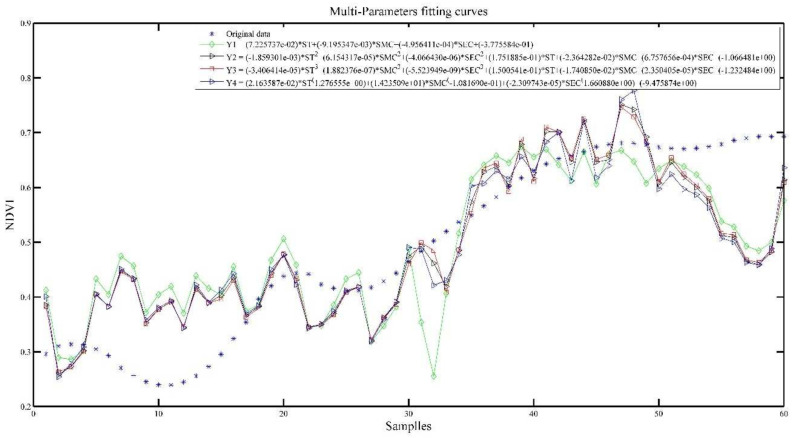
IMP and NDVI model results.

**Figure 11:**
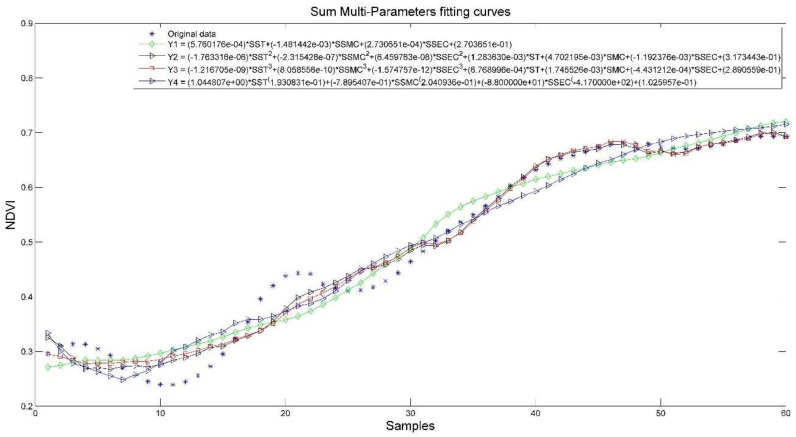
SIMP and NDVI model results.

Figure 10 and Figure 11 show the effect of integration of multiple parameters (IMP) and sum of integration of multiple parameters (SIMP) with NDVI fit, respectively.

In Table 6, the R^2^ of the IMP and SIMP are improved, and the best ones reach above 0.6. The coefficients of the models after fusing the three parameters (cumulative) all reach above 0.9, and the best one reaches 0.9761, which indicates that the prediction effect is more satisfactory.

For IMP model, Y3 model has the best performance. For SIMP model, Y2 model has the best performance. The trend of NDVI is well represented in both models.

#### 3.3.5. Model Prediction Performance Validation

Validation experiments were performed on the model mentioned above. Its performance was experimented on each model using 12 sets of data. Indicators for the evaluation of the model are MSE, RMSE, R^2^ and AR.

Table 7, Table 8, Table 9 and Table 10 show the results of the validation experiments.

In Table 7, the R^2^ of each model is greater than 0.8 when the model input is ST and above 0.95 when the input is SST. The performance of the Y3 model is the worst among all the models. Furthermore, in Table 8, the Y3 model predicts best when the model input is SMC, while the Y2 model predicts best when the model input is SSMC.

In Table 9, the Y3 model has the best prediction performance when the model input is SEC, and the R^2^ of the model is 0.8059, while the R^2^ of the model prediction is above 0.95 when the model input is SSEC, which indicates a better prediction performance.

In Table 10, when the model input is IMP, R^2^ of Y1 and Y3 model are 0.8179 and 0.8051, which is obviously better than other models. When the model input is SIMP, R^2^ of Y1 and Y4 model are 0.8017 and 0.9308, which is obviously better than Y2 and Y3 models.

This is because the short duration of the experiment and the inconsistent details of the collection of soil and NDVI data can affect the results obtained. The impact of the mathematical manipulation of the data to obtain the final regressions in the polynomial models may also be relevant. Therefore, the performance of the constructed model and the model validation performance need to be considered together to determine which model is more suitable.

#### 3.3.6. Summary

A tea tree growth monitoring model was constructed. Prediction models based on soil temperature, soil moisture content and soil electrical conductivity with NDVI were constructed. Five prediction models were constructed using the polynomial model and the power function model, among which the cubic polynomial fit was the best and the evaluation parameters were optimal, which were consistent with the physiological process of tea tree growth. The performance of various models with different input characteristics of the model is analyzed. A novel idea of tea tree growth monitoring model construction is given. The short experimental period and the disparity of detail in the collection of soil and NDVI data may affect the results obtained.

## 4. LSTM Networks Model Optimized by BES

### 4.1. LSTM

**Figure 12:**
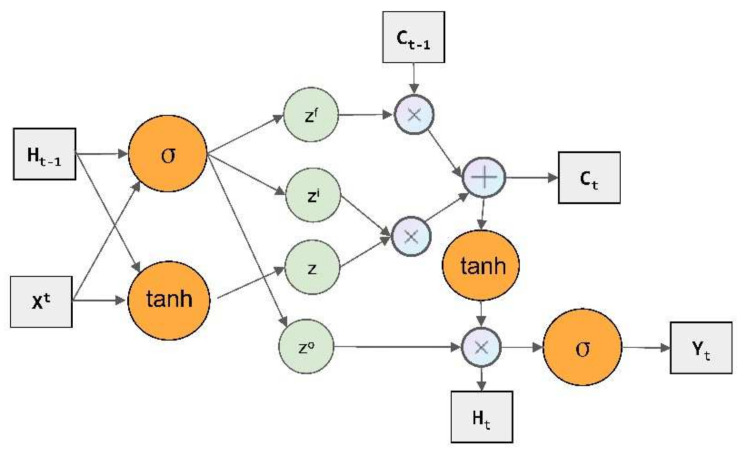
LSTM structure.

LSTM network is a special form of Recurrent Neural Network (RNN) [30], which mainly consists of the forgetting phase, selecting memory phase and output phase, with strong generalization ability. It can effectively solve the gradient disappearance and gradient explosion problems during the training of long sequences and performs better in the prediction of nonlinear long sequences. The LSTM structure is shown in Figure 12, where ⊗ is matrix multiplication, ⊕ is matrix summation; $z^{i}$, $z^{f}$ and $z^{o}$ are the gate control signal; z is a transformed value using the tanh activation function (takes values in the range [−1, 1]); and $\;H_{t-1}$, $c^{t-1}$, $H^{t}$ and $c^{t}$ are the previous moment state value and the current moment state value of the two transmission states. σ is the sigmoid activation function, $X^{t}$ is input signal and $Y^{t}$ is output signal. They are calculated by Equations (7)–(13).

(7) $$z=\tanh(W,X^{t},H_{t-1})$$

(8) $$z^{i}=\sigma (W^{i},X^{t},H_{t-1})$$

(9) $$z^{f}=\sigma (W^{f},X^{t},H_{t-1})$$

(10) $$z^{o}=\sigma (W^{o},X^{t},H_{t-1})$$

(11) $$c^{t}=z^{f}⊗c^{t-1}⊕z^{i}⊗z$$

(12) $$H^{t}=z^{o}⊗\tanh\left(c^{t}\right)$$

(13) $$Y^{t}=\sigma \left(W^{’}H^{t}\right)$$

### 4.2. BES

BES is an algorithm that has powerful global search capability and can effectively solve various complex numerical optimization problems. It consists of three phases, which are the selection phase, search phase and prey phase [31,32].

In the selection phase, the bald eagle first selects the area at random and then looks for the best position by judging the prey population. At this stage, the position of the main bald eagle is determined primarily by experience and position change parameters, as shown in Equation (14).

(14) $$P_{i,\mathrm{new}}=P_{\mathrm{best}}+\alpha \times \gamma \times (P_{\mathrm{mean}}-P_{i})$$

where α  represents the position change control parameter that takes a value between 1.5 and 2, γ is a random number between 0 and 1, $P_{\mathrm{best}}$ represents when the bald eagle has the best search position identified during its previous search, $P_{\mathrm{mean}}$ indicates that these eagles have used up all information from the previous points and $P_{i}$ is the position of the i-th bald eagle.

In the search phase, the bald eagle is in search of prey to speed up the search process and find the best swooping location. At this point, the best position for the eagle is determined by Equation (15).

(15) $$P_{i,\mathrm{new}}=P_{i}+x\left(i\right)\times \left(P_{i}-P_{\mathrm{mean}}\right)+y\left(i\right)\times \left(P_{i}-P_{i+1}\right)\;\;x\left(i\right)\;=\frac{xr\left(i\right)}{\max\left(\left|xr\right|\right)}\;y\left(i\right)\;=\frac{yr\left(i\right)}{\max\left(\left|yr\right|\right)}\;\;xr\left(i\right)\;=r\left(i\right)\times \sin\left(\theta \left(i\right)\right)\;yr\left(i\right)\;=r\left(i\right)\times \cos\left(\theta \left(i\right)\right)\;\;r\left(i\right)\;=\theta \left(i\right)+R\times rand\;\theta \left(i\right)\;=a\times \pi \times rand$$

where $\theta \left(i\right)$ and $r\left(i\right)$ represent the polar angle and polar diameter of the spiral equation; φ is a parameter determining the corner between the point search in the central point, which takes a value between 5 and 10; *R* is a parameter determining the number of search cycles that takes a value between 0.5 and 2; rand is a random number from 0 to 1; and $x\left(i\right)\;$ and $y\left(i\right)$ indicate the position of the bald eagle in polar coordinates.

In the swooping stage, bald eagles swoop quickly from the best spots in the search space to the target, while individuals from other species move to the best spots and attack the prey. The position of the bald eagle at this point is calculated using Equation (16).

(16) $$P_{i,\mathrm{new}}=\mathrm{rand}\times P_{\mathrm{best}}+x1\left(i\right)\times \left(P_{i}-c1\times P_{\mathrm{mean}}\right)+y1\left(i\right)\times \left(P_{i}-c2\times P_{\mathrm{best}}\right)\;\;x1\left(i\right)\;=\frac{xr\left(i\right)}{\max\left(\left|\mathrm{xr}\right|\right)}\;y1\left(i\right)\;=\frac{\mathrm{yr}\left(i\right)}{\max\left(\left|\mathrm{yr}\right|\right)}\;\;\mathrm{xr}\left(i\right)\;=r\left(i\right)\times \sin h\left(\theta \left(i\right)\right)\;\mathrm{yr}\left(i\right)\;=r\left(i\right)\times \cos h\left(\theta \left(i\right)\right)\;\;\theta \left(i\right)\;=a\times \pi \times \mathrm{rand}\;r\left(i\right)\;=\theta \left(i\right)$$

where *c*1 and *c*2 represent the exercise intensity of the bald eagle to the best and center position that takes the value between 1 and 2.

### 4.3. Algorithm Optimization

The initialization parameters directly affect prediction performance the LSTM networks, and parameter optimization is of particular important. The learning rate and the number of hidden layers are important parameters in LSTM networks, which are optimized by taking advantage of the optimization ability, convergence ability and convergence speed of the BES algorithm to avoid the influence of human empirical differences. The tuning steps are as follows:

(1) Process the data and split it into training set and test set.
(2) Initializing the parameters of the BES and the LSTM networks.
(3) Obtain the best parameters using the BES to set up the LSTM network.
(4) The RMSE of the model is used as the fitness, and the fitness of each population in the BES is calculated, and the minimum value is taken as the optimal solution result.
(5) Iterate the operation and update the parameters of the LSTM using the BES algorithm.
(6) Repeat (4)–(5) until the end of the condition.
(7) Prediction of the LSTM network using the final optimized parameters.

### 4.4. Evaluation Indicators

The indicators used for the model predictions are $R^{2}$, RMSE, MSE, MAE and MAPE.

### 4.5. Experimental Analysis

**Figure 13:**
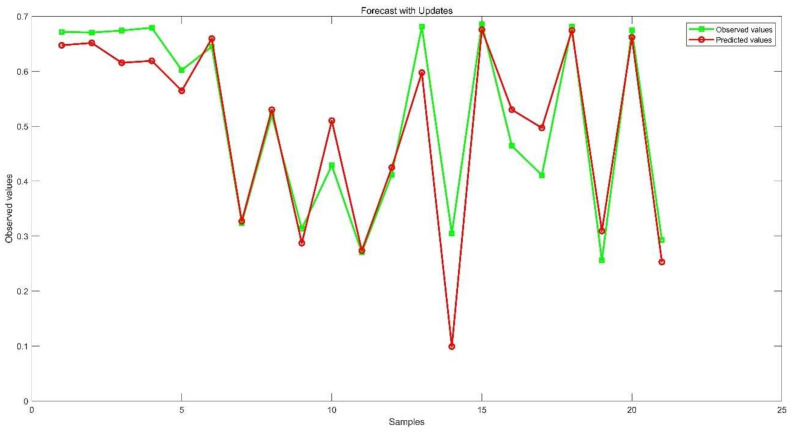
LSTM model prediction results.

The first 50 sets of data were used as the training set, and the remaining 21 sets were used as the test set. Then, the LSTM network and BES–LSTM were used for training and testing, and the results are shown in Figure 13 and Table 11.

Figure 11 shows that the values provided for in the BES–LSTM network all capture the trend of NDVI values. The R^2^, RMSE, MSE, MAE and MAPE of the LSTM model are 0.5299, 0.1642, 0.0270, 0.1518 and 36.8900, respectively, while the evaluation indicators of the BES–LSTM model are 0.8666, 0.0629, 0.0040, 0.0436 and 10.5257, respectively. The BES–LSTM model is significantly better than the LSTM network, indicating that the BES–LSTM is a better-performing model.

### 4.6. Summary

A tea tree growth monitoring model based on LSTM optimized by BES was constructed. The principles of LSTM and BES algorithm were explained, the learning rate and the number of hidden layers of the LSTM network were optimized using the BES algorithm and the optimized BES–LSTM network was used to build a tea tree growth monitoring model, which effectively improved the performance of the network prediction. In this model, the parameters such as soil temperature, soil moisture content and soil electrical conductivity were used as input values, and NDVI was used as output value, which could effectively capture the trend of NDVI. The R^2^, RMSE, MSE, MAE and MAPE of the LSTM model were 0.5299, 0.1642, 0.0270, 0.1518 and 36.8900, respectively, while those of the BES–LSTM model were 0.8666, 0.0629, 0.0040, 0.0436 and 10.5257, respectively, which indicates that the performance of BES–LSTM is far better than that of LSTM.

## 5. Conclusions

Our work is summarized in the present section. A tree growth monitoring model constructed with soil indicators was proposed. The correlation of soil temperature, soil moisture content and soil electrical conductivity with the tea tree growth parameter (NDVI) was discussed. The results showed that the NDVI value was positively correlated with soil temperature, while it was inversely correlated with soil moisture content and soil electrical conductivity. The results also showed that it was positively correlated with sum of soil temperature, sum of soil moisture content and sum of soil electrical conductivity.

The final regression results in the polynomial model with discrete input characteristics are discussed, which proposes an innovative method for a tea tree growth monitoring model. The performance of the model was further verified. The experiments showed that the tea tree growth monitoring model achieved a better prediction effect.

A prediction model based on the LSTM model optimized by arithmetic optimization algorithm (AOA) was investigated, providing a new idea for tea growth monitoring. In this model, parameters such as soil temperature, soil moisture content and soil electrical conductivity were used as input values, and NDVI was used as output value. The experiments show that the AOA–LSTM model predicts a value of 0.8666, which has a certain advantage over the performance of other models. The model proposed in this chapter provides a different idea for tea growth monitoring.

Some issues in the paper need to be further discussed:

(1) The tea tree growth monitoring model needs to be strengthened. As tea tree growth monitoring is more influenced by other factors in the environment, it has yet to be further studied in depth. The relationship between tea tree growth monitoring and the influence of meteorological parameters, atmosphere, etc., should be effectively studied and integrated into the model to achieve better results.
(2) More effective network model applications are expected. Since the impact of each parameter in the soil information is also mutual, more complex networks need to be used to build models that can better predict and improve the accuracy of prediction.
(3) Data samples need to be increased. The remote sensing data used in this study are only of one year, and only 72 remote sensing images can be extracted to extract NDVI, this making the data applied to the experiment obviously less. More NDVI data should be obtained, and the period of the experiment should be extended, so that the accuracy and applicability of the prediction model can be improved.

## Figures and Tables

**Table 1:** Parameters of NECMEC20 Sensor.

Indicators	Range	Resolution	Precision
SMC	0~50% 0~100%	0.03% 1%	2% (0~50%) 3% (50~100%)
SEC	0–5000 μs/cm 10,000 μs/cm 20,000 μs/cm	10 μs/cm 50 μs/cm 50 μs/cm	±3% ±5% ±5%
ST	−40~80 °C	0.1 °C	±0.5 °C

**Table 2:** Selected data of soil information of tea plantation.

No.	ST/°C	SMC/%	SEC/μs/cm	Time
1	12.76	18.83	60	2020-12-31 23:51:31
2	12.85	18.87	60	2020-12-31 23:40:33
3	12.85	18.83	60	2020-12-31 23:30:30
4	12.85	18.83	60	2020-12-31 23:19:35
5	12.88	18.87	60	2020-12-31 23:08:32
6	12.88	18.83	60	2020-12-31 22:57:35
7	12.91	18.87	60	2020-12-31 22:47:32
8	12.97	18.87	60	2020-12-31 22:36:35
9	12.97	18.87	60	2020-12-31 22:26:32
10	12.97	18.87	60	2020-12-31 22:15:35

**Table 3:** Evaluation of the temperature construction model.

Item	Model	SSE	RMSE	$R^{2}$	AR
ST	Y1	0.6730	0.1077	0.5673	0.5599
	Y2	0.6654	0.1080	0.5723	0.5573
	Y3	0.5649	0.1004	0.6369	0.6173
	Y4	0.6732	0.1077	0.5672	0.5598
	Y5	0.6704	0.1085	0.5690	0.5539
SST	Y1	0.1111	0.0438	0.9286	0.9274
	Y2	0.0815	0.0378	0.9476	0.9458
	Y3	0.0563	0.0317	0.9638	0.9619
	Y4	0.1642	0.0532	0.8945	0.8926
	Y5	0.1000	0.0419	0.9357	0.9334

**Table 4:** Evaluation of the soil moisture content construction model.

Item	Model	SSE	RMSE	$R^{2}$	AR
SMC	Y1	1.0757	0.1249	0.3916	0.3828
	Y2	1.0684	0.1253	0.3958	0.3780
	Y3	0.7455	0.1055	0.5784	0.5595
	Y4	1.1734	0.1304	0.3364	0.3268
	Y5	1.0757	0.1258	0.3916	0.3738
SSMC	Y1	0.1959	0.0533	0.8892	0.8876
	Y2	0.1803	0.0515	0.8980	0.8950
	Y3	0.0601	0.0300	0.9660	0.9645
	Y4	0.2711	0.0627	0.8467	0.8445
	Y5	0.1946	0.0535	0.8900	0.8867

**Table 5:** Evaluation of the soil electrical conductivity construction model.

Item	Model	SSE	RMSE	$R^{2}$	AR
SEC	Y1	1.0757	0.1249	0.3916	0.3828
	Y2	1.0684	0.1253	0.3958	0.3780
	Y3	0.7455	0.1055	0.5784	0.5595
	Y4	1.1734	0.1304	0.3364	0.3268
	Y5	1.0757	0.1258	0.3916	0.3738
SSEC	Y1	0.1959	0.0533	0.8892	0.8876
	Y2	0.1803	0.0515	0.8980	0.8950
	Y3	0.0601	0.0300	0.9660	0.9645
	Y4	0.2711	0.0627	0.8467	0.8445
	Y5	0.1946	0.0535	0.8900	0.8867

**Table 6:** Evaluation of the integration of multiple parameters model.

Item	Model	SSE	RMSE	$R^{2}$	AR
IMP	Y1	0.6619	0.1050	0.5745	0.5672
	Y2	0.5401	0.0949	0.6528	0.6468
	Y3	0.5281	0.0938	0.6605	0.6547
	Y4	0.5750	0.0979	0.6305	0.6241
SIMP	Y1	0.0695	0.0340	0.9553	0.9545
	Y2	0.0372	0.0249	0.9761	0.9757
	Y3	0.0435	0.0269	0.9720	0.9715
	Y4	0.0676	0.0336	0.9565	0.9558

**Table 7:** Validation evaluation of the temperature construction model.

Item	Model	MSE	RMSE	$R^{2}$	AR
ST	Y1	0.0745	0.2730	0.8907	0.8785
	Y2	0.0741	0.2722	0.8918	0.8798
	Y3	0.0837	0.2893	0.8281	0.8090
	Y4	0.0738	0.2717	0.8906	0.8785
	Y5	0.0742	0.2724	0.8912	0.8791
SST	Y1	0.0299	0.1728	0.9902	0.9891
	Y2	0.0097	0.0986	0.9906	0.9896
	Y3	0.0005	0.0226	0.9739	0.9710
	Y4	0.0098	0.0989	0.9907	0.9897
	Y5	0.0217	0.1472	0.9904	0.9893

**Table 8:** Validation evaluation of the soil moisture content construction model.

Item	Model	MSE	RMSE	$R^{2}$	AR
SMC	Y1	0.0295	0.1718	0.5410	0.4900
	Y2	0.1230	0.3507	0.5559	0.5066
	Y3	0.0504	0.2244	0.6218	0.5798
	Y4	0.1347	0.3671	0.5757	0.5286
	Y5	0.1123	0.3351	0.5706	0.5229
SSMC	Y1	0.0075	0.0867	0.9749	0.9721
	Y2	0.0027	0.0522	0.9858	0.9842
	Y3	0.00006	0.0080	0.9341	0.9268
	Y4	0.0036	0.0599	0.9768	0.9743
	Y5	0.0058	0.0764	0.9757	0.9729

**Table 9:** Validation evaluation of the soil electrical conductivity construction model.

Item	Model	SSE	RMSE	$R^{2}$	AR
SEC	Y1	0.0033	0.0571	0.6807	0.6452
	Y2	0.0046	0.0680	0.6879	0.6532
	Y3	0.0002	0.0126	0.8059	0.7843
	Y4	0.0055	0.0739	0.7352	0.7058
	Y5	0.0033	0.0575	0.6812	0.6458
SSEC	Y1	0.0056	0.0747	0.9778	0.9753
	Y2	0.0033	0.0575	0.9798	0.9776
	Y3	0.0003	0.0192	0.9532	0.9479
	Y4	0.0027	0.0517	0.9788	0.9764
	Y5	0.0051	0.0713	0.9779	0.9755

**Table 10:** Validation evaluation of the integration of multiple parameters model.

Item	Model	MSE	RMSE	$R^{2}$	AR
IMP	Y1	0.0266	0.1630	0.8179	0.8148
	Y2	0.0078	0.0885	0.7149	0.7100
	Y3	0.0237	0.1540	0.8051	0.8017
	Y4	0.0298	0.1727	0.3282	0.3166
SIMP	Y1	0.0053	0.0727	0.8017	0.7983
	Y2	0.0002	0.0168	0.6594	0.6535
	Y3	0.0057	0.0752	0.2617	0.2492
	Y4	0.0103	0.1013	0.9308	0.9296

**Table 11:** Evaluation of LSTM model and BES–LSTM model.

Model	$R^{2}$	RMSE	MSE	MAE	MAPE
LSTM	0.5299	0.1642	0.0270	0.1518	36.8900
BES-LSTM	0.8666	0.0629	0.0040	0.0436	10.5257

## Data Availability

Not applicable.
